# Neurogenetic and multi‐omic sources of overlap among sensation seeking, alcohol consumption, and alcohol use disorder

**DOI:** 10.1111/adb.13365

**Published:** 2024-01-29

**Authors:** Alex P. Miller, Ian R. Gizer

**Affiliations:** ^1^ Department of Psychiatry Washington University School of Medicine St. Louis Missouri USA; ^2^ Department of Psychological Sciences University of Missouri Columbia Missouri USA

**Keywords:** alcohol consumption, alcohol use disorder, genetics, genomic structural equation modelling, multi‐omics, neuroimaging, sensation seeking

## Abstract

Sensation seeking is bidirectionally associated with levels of alcohol consumption in both adult and adolescent samples, and shared neurobiological and genetic influences may in part explain these associations. Links between sensation seeking and alcohol use disorder (AUD) may primarily manifest via increased alcohol consumption rather than through direct effects on increasing problems and consequences. Here the overlap among sensation seeking, alcohol consumption, and AUD was examined using multivariate modelling approaches for genome‐wide association study (GWAS) summary statistics in conjunction with neurobiologically informed analyses at multiple levels of investigation. Meta‐analytic and genomic structural equation modelling (GenomicSEM) approaches were used to conduct GWAS of sensation seeking, alcohol consumption, and AUD. Resulting summary statistics were used in downstream analyses to examine shared brain tissue enrichment of heritability and genome‐wide evidence of overlap (e.g., stratified GenomicSEM, RRHO, genetic correlations with neuroimaging phenotypes), and to identify genomic regions likely contributing to observed genetic overlap across traits (e.g., H‐MAGMA and LAVA). Across approaches, results supported shared neurogenetic architecture between sensation seeking and alcohol consumption characterised by overlapping enrichment of genes expressed in midbrain and striatal tissues and variants associated with increased cortical surface area. Alcohol consumption and AUD evidenced overlap in relation to variants associated with decreased frontocortical thickness. Finally, genetic mediation models provided evidence of alcohol consumption mediating associations between sensation seeking and AUD. This study extends previous research by examining critical sources of neurogenetic and multi‐omic overlap among sensation seeking, alcohol consumption, and AUD which may underlie observed phenotypic associations.

## Abstract 2

Sensation seeking is associated with greater alcohol consumption which, in turn, can increase risk for alcohol use disorder. We found substantial overlap of neurogenetic and multi‐omic influences underlying sensation seeking and alcohol consumption and evidence that alcohol consumption mediates genetic links between sensation seeking and alcohol use disorder.

## 1 INTRODUCTION

Sensation seeking, the tendency to prefer and engage in intense, novel, and rewarding activities and experiences,^1^ is associated with quantity and frequency of alcohol consumption,^2^, ^3^ often more so than the negative alcohol‐related consequences characteristic of alcohol use disorder (AUD). Further, the association between sensation seeking and alcohol consumption appears to be both developmentally relevant and bidirectional. Specifically, higher sensation seeking in adolescence is associated with earlier alcohol use initiation and greater increases in prospective heavy drinking.^4^, ^5^ In turn, higher levels of consumption predict subsequently higher levels of sensation seeking, potentially exacerbated by neurobiological changes resulting from heavy alcohol use in adolescence.^6^, ^7^

Competing theories explaining the relation between sensation seeking and alcohol consumption have been described in the literature. Broadly, dual‐systems models contend that bottom‐up reward‐based drive (i.e., sensation seeking) leads to early consumption and lack of top‐down self‐regulation leads to the development of heavier alcohol consumption and subsequent consequences.^8^, ^9^ In contrast, acquired preparedness models argue that associations between sensation seeking and alcohol consumption are partially mediated by enhancement drinking motives and positive expectancies.^10^ Despite their differences, both models posit that early drinking is driven by the positively reinforcing effects of alcohol and suggest links to later AUD development may be indirect, such that sensation seeking leads to increases in consumption that, in turn, increase risk for AUD.

Extensive theory and empirical testing point to models of shared neurobiology underlying relations among sensation seeking, alcohol consumption, and AUD. For example, the ‘addiction cycle’ model argues that the initial binge/intoxication stage of the cycle is primarily driven by bottom‐up incentive‐reward systems localised to the basal ganglia and midbrain and impaired top‐down control of these approach systems by the prefrontal cortex linked via mesocorticolimbic pathways.^11^ AUD progression follows from continuation of the cycle with withdrawal/negative affect (extended amygdala) and preoccupation/anticipation stages (i.e., craving; prefrontal cortex, insula) signalling a shift in the relative influence of the neurobiological circuits contributing to continued drinking behaviours. Neuroimaging studies provide corroborating evidence of some of these hypothesised addiction pathways^12^ and demonstrate overlap between alcohol consumption and sensation seeking with respect to regional brain morphology and functional connectivity. For example, studies have demonstrated associations between sensation seeking and neuroanatomical differences in frontocingulate thickness and surface area, volume of basal ganglia structures (e.g., nucleus accumbens and globus pallidus),^13^, ^14^ and reward‐cued connectivity of frontostriatal pathways,^15^, ^16^ highlighting many of the same regions and circuits associated with the described binge/intoxication stage. As some of these observed differences extend beyond hypothesised subcortical regions used to characterize sensation seeking as a ‘bottom‐up’ process and clearly implicate relevant cortical features,^13^, ^17^, ^18^ cortical neuroimaging phenotypes (e.g., regional volume, thickness, and surface area), including phenotypes assessing cortical–subcortical connections, may help to explain relations between sensation seeking and alcohol use phenotypes at the neurobiological level.

Consistent with models suggesting that alcohol consumption and AUD are driven, in part, by separable biological underpinnings, molecular genetics research has revealed separable genetic influences contributing to each phenotype despite substantial overlap between them, suggesting that alcohol consumption has limitations as a direct genetic proxy for AUD.^19^, ^20^ Thus, investigations examining differences in genetic associations among sensation seeking, alcohol consumption, and AUD are warranted. Prior evidence suggests that sensation seeking may be more strongly genetically correlated with alcohol consumption than AUD,^19^, ^21^ but direct comparisons of these relative associations have not been investigated. Further, investigations attempting to further elucidate the nature of the neurogenetic (i.e., genetic influences on individual differences in brain function) pathways linking sensation seeking, alcohol consumption, and AUD using large‐scale genomic data and advanced post‐multivariate genome‐wide association study (GWAS) approaches have not yet been performed.

Given that such neurogenetic investigations have the potential to further refine aetiological and theoretical frameworks of AUD development, the current study aimed to identify shared and unique sources of genetic overlap among sensation seeking, alcohol consumption, and AUD using large‐scale genomic, neuroimaging, transcriptomic, epigenomic, and chromatin‐based data. Multiple statistical approaches, spanning different levels of genetic analysis (i.e., single‐variant and gene‐based), were used to assess neurobiological sources of overlap among these traits. It was hypothesised that, across analytic approaches, sensation seeking would demonstrate stronger associations with alcohol consumption than with AUD, that genetic associations between sensation seeking and AUD would be partially mediated by alcohol consumption, and that neurogenetic evidence of overlap among traits would map onto brain regions implicated by previous theory and research as summarised earlier in the text.

## 2 METHODS

Descriptions of GWAS used in this study are presented in Table 1. GWAS were restricted to individuals of European ancestry and single nucleotide polymorphisms (SNPs) with minor allele frequencies (MAF) > 0.01. See Supporting Information Methods for descriptions of genotyping, imputation, quality control, and additional phenotypic measurement information for GWAS used.

**TABLE 1:** Overview of GWAS used in study.

GWAS phenotypes	GWAS sample size	Number of SNPs(MAF > 0.01)	Sample/cohort
Sensation seeking	710,971^a^	7,582,355	
Adventurousness	557,928	9,151,591	23andMe^22^
Risk taking	490,873	9,520,439	23andMe + UKB + 10 rep. samples^22^
UPPS‐P sensation seeking	22,745	9,006,418	23andMe^21^
Alcohol consumption	1,388,120^a^	7,019,598	
Drinks per week	941,288	10,310,172	GSCAN^23^
AUDIT‐C	141,932	10,108,726	UKB + MVP^24^, ^25^
Grams of alcohol per day	480,843	7,703,032	UKB + AlcGen + CHARGE Plus^26^
Alcohol use disorder	220,182^b^	9,533,157	
Alcohol dependence	26,853^b^	8,507,344	PGC^27^
Alcohol use disorder	152,332^b^	5,068,448	MVP^28^
Alcohol use disorder	40,997^b^	8,720,146	FinnGenR6^29^
Neuroimaging			
Cortical volume			UKB^30^
31 right + 31 left hemisphere regions	31,968	9,279,434	
Cortical surface area and thickness			ENIGMA + UKB^31^
Surface area (34 regions)	33,992	8,376,876	
Thickness (34 regions)	33,992	8,357,547	
Sub‐cortical volume			CHARGE + ENIGMA + UKB^32^
Nucleus accumbens	28,697	7,563,415	
Amygdala	30,142	7,066,805	
Brainstem	24,945	7,049,063	
Caudate nucleus	30,153	6,778,919	
Globus pallidus	30,124	7,601,584	
Putamen	29,984	6,785,509	
Thalamus	30,175	7,609,352	
rs‐fMRI network connectivity	34,691	9,025,926	UKB^33^
124 functional connectivity phenotypes			

Abbreviations: AlcGen = Alcohol Genome‐Wide Consortium; CHARGE = Cohorts for Heart and Aging Research in Genomic Epidemiology; ENIGMA = Enhancing NeuroImaging Genetics through Meta‐Analysis Consortium; FinnGenR6 = FinnGen Research Project Release 6; GSCAN = GWAS and Sequencing Consortium of Alcohol and Nicotine use; MAF = minor allele frequency; MVP = Million Veterans Program; PGC = Psychiatric Genomics Consortium; rs‐fMRI = resting‐state functional magnetic resonance imaging; UKB = UK Biobank.

^a^ $\hat{N}$ = GenomicSEM calculated effective sample size for latent factors.

^b^ *n*_effective_ = effective sample size for case/control GWAS meta‐analyses.

### 2.1 GWAS

As described previously,^18^ GWAS of three phenotypes (adventurousness,^22^ risk taking,^22^ and UPPS‐P sensation seeking^21^) conducted using primarily 23andMe, Inc., and UK Biobank (UKB) samples were specified as indicators of a sensation seeking genomic factor. Three GWAS were specified as indicators of an alcohol consumption genomic factor: (1) a ‘drinks per week’ GWAS meta‐analysis using data from 23andMe (*N* = 403,939) and the GWAS and Sequencing Consortium of Alcohol and Nicotine use (GSCAN; *N* = 537,341)^23^; (2) an AUDIT‐C GWAS meta‐analysis using data from UKB (*N* = 121,604)^24^ and Million Veteran Program (MVP) cohorts (*N* = 200,680)^25^; and (3) an existing GWAS meta‐analysis of grams of alcohol consumed per day using UKB, the Alcohol Genome‐Wide (AlcGen) Consortium, and the Cohorts for Heart and Aging Research in Genomic Epidemiology Plus (CHARGE+) Consortium.^26^ For AUD, a GWAS meta‐analysis was conducted using summary statistics for AUD/alcohol dependence from the Psychiatric Genomics Consortium (PGC),^27^ the FinnGen Research Project Release 6 (FinnGenR6),^29^ and MVP.^28^

Four sets of neuroimaging GWAS were utilised for genetic correlation analyses: (1) 62 bilateral cortical parcellation phenotypes^30^; (2) 34 cortical surface area and thickness parcellation phenotypes^31^; (3) volumes of seven subcortical structures^32^; and (4) 124 resting‐state functional magnetic resonance imaging (rs‐fMRI) functional connectivity phenotypes.^33^

### 2.2 Data analysis

#### 2.2.1 GWAS meta‐analyses

METAL^34^ was used to conduct sample size‐weighted GWAS meta‐analyses of non‐overlapping cohort‐level summary statistics for all described meta‐analyses. Genetic correlations confirmed uniformly high concordance between each sample for each phenotype, justifying the meta‐analytic approach (Table S1; see Figure S1 for quantile‐quantile [*Q*‐*Q*] plots). Reported effective sample sizes for MVP and PGC and calculated FinnGenR6 effective sample size were used for weighting in the AUD meta‐analysis.

#### 2.2.2 Genomic factor analysis using GenomicSEM

GenomicSEM (v0.0.5)^35^ was employed using diagonally weighted least squares estimation and unit variance identification to conduct a correlated three‐factor confirmatory analysis modelling genetic associations among sensation seeking, alcohol consumption, and AUD. Notably, GenomicSEM adjusts for sample overlap by estimating a sampling covariance matrix that indexes the extent to which sampling errors of the estimates are associated.^35^ AUD was modelled as a dummy latent factor by specifying a loading of 1 and 0 residual variance to allow for its inclusion in this model. Model fit was assessed using *χ* ^2^, comparative fit index (CFI), standardised root mean square residual (SRMR), and Akaike information criterion (AIC) values.

Stratified GenomicSEM^36^ analyses were then conducted to examine differential and shared enrichment of sensation seeking and alcohol consumption genomic factor variances and their covariance within multiple brain‐related functional annotations. Specifically, a model was fit allowing the variances of each factor and the covariance between them to vary across annotations to test for enrichment of these parameters. Functional annotations included (1) baseline annotations from 1000 Genomes Project Phase 3 (BaselineLD v2.2)^37^; (2) annotations for tissue‐specific epigenomic marks across seven brain regions and five different post‐translational modifications (H3K36me3, H3K4me1, H3K4me3, H3K9ac and H3K27ac) using data from the Roadmap Epigenomics Consortium^38^; and (3) annotations for tissue‐specific gene expression across 13 brain regions constructed using RNA sequencing data from the Genotype‐Tissue Expression project version 8 (GTEx v8).^39^ GTEx v8 annotations were constructed using stratified linkage disequilibrium score regression (LDSC) applied to specifically expressed genes (LDSC‐SEG; Supporting Information Methods).^40^ In total, enrichment analyses were based on 97 binary annotations. Tissue‐specific heritability enrichment analysis of AUD was conducted using LDSC‐SEG. A 5% false discovery rate (FDR) correction was used within each model parameter (i.e., variance/covariance of GenomicSEM factors and heritability of AUD) to account for multiple testing.

#### 2.2.3 Multivariate GWAS

Multivariate GWAS were conducted to estimate SNP associations with sensation seeking and alcohol consumption latent factors separately (i.e., single factor models) using GenomicSEM (see Supporting Information Methods). Individual effects for SNPs available across all indicator GWAS and present in the 1000 Genomes Project Phase 3 v5 reference panel^41^ were estimated for each trait. Effective sample sizes ($\hat{N}$) were estimated using the approach described by Mallard et al.^42^ SNP‐based heritability estimates ($h_{g}^{2}$) for latent genomic factors are more accurately referred to as *genetic variances*, and thus are subsequently denoted by *ζ*_*g*_. Follow‐up multivariate GWAS that included unique pathways from SNPs to each indicator were conducted to calculate *Q*_SNP_ heterogeneity tests.^35^ SNPs with genome‐wide significant (GWS) *Q*_SNP_ statistics (*P* < 5 × 10^−8^) reflect associations not fully mediated by the latent genomic factor (i.e., common pathway model). These were removed from downstream analyses to reduce heterogeneity (Figure S2).

#### 2.2.4 Post‐multivariate GWAS analyses

##### Mediation model

A follow‐up mediation model was examined in GenomicSEM using AUD and sensation seeking and alcohol consumption latent factor summary statistics. This model tested whether genetic influences underlying sensation seeking exert direct effects on AUD or whether this relation is mediated by effects on alcohol consumption, which in turn influence AUD (indirect path).

##### Gene‐based analyses

Multi‐marker Analysis of GenoMic Annotation (MAGMA, v1.08)^43^ via FUMA (v1.3.7)^44^ was used to conduct gene‐based association tests. SNPs were mapped to protein‐coding genes using Ensembl build 92. Chromatin interaction mapping was performed via Hi‐C‐coupled MAGMA (H‐MAGMA)^45^ using MAGMA v1.10 and two Hi‐C datasets obtained from the Won Lab GitHub repository (https://github.com/thewonlab/H-MAGMA ): (1) cortical neurons from the dorsolateral prefrontal cortex (dlPFC) and (2) dopaminergic neurons from the midbrain (DA‐midbrain), including the ventral tegmental area and substantia nigra. H‐MAGMA extends MAGMA by incorporating long range chromatin interaction profiles (Hi‐C) to identify neurobiologically relevant SNP‐gene‐tissue associations for non‐coding (intergenic and intronic) SNPs. Significant MAGMA and H‐MAGMA genes were identified using Bonferroni‐corrected one‐sided *P*‐values adjusted for the number of genes tested.

Gene‐level association statistics obtained from H‐MAGMA were used to compute rank–rank hypergeometric tests of overlap (RRHO), a threshold‐free algorithm for comparing two genomic datasets theoretically less sensitive to differences in sample size,^46^ using the *RRHO* R package (v1.38.0).^47^ Ranked gene lists for each trait ordered by corresponding H‐MAGMA *z*‐statistics were compared to quantify gene‐level overlap between each pair of traits, adjusting for multiple comparisons using the Benjamini and Yekutieli FDR (BY FDR).^48^ *Z*‐scores computed from maximum BY FDR‐corrected‐log_10_ *P*‐values were used to characterise overlap. RRHO output‐identified upregulated genes for each pairwise trait comparison within each annotation set were defined as pleiotropic.^45^

##### Local genetic correlation analyses

Using local analysis of covariant association (LAVA),^49^ univariate local genetic signals (local $h_{g}^{2}$) were examined within each trait across 2,945 partitioned semi‐independent genomic regions of ~1 Mb. Regions demonstrating univariate signals for more than one trait following Bonferroni correction (*P* < 2.00 × 10^−5^) were selected for tests of local genetic correlation (*ρ*_*g*_). This resulted in 1,608 bivariate tests, with significant local genetic correlations among sensation seeking, alcohol consumption, and AUD identified by a second Bonferroni correction (*P* < 3.11 × 10^−5^).

##### Neuroimaging genetic correlation analyses

A series of LDSC genetic correlation analyses were conducted in GenomicSEM to examine whether patterns of genetic correlations with regional cortical volume, surface area, thickness, subcortical structure volume, and resting‐state connectivity phenotypes differed across sensation seeking, alcohol consumption, and AUD. A 5% FDR correction was used to account for multiple testing within each neuroimaging phenotype set. To determine whether correlations with each neuroimaging phenotype differed across traits, *χ* ^2^ tests, using the approach described by Demange et al.,^50^ were used to evaluate the null hypotheses that (A) pairs of genetic correlations or (B) all correlations with each neuroimaging phenotype could be constrained to equality. Follow‐up *Q*_trait_ analyses were conducted to examine heterogeneity in associations between indicator GWAS and neuroimaging phenotypes not well accounted for by latent genomic factors.^51^ Similar to *Q*_SNP_ analyses, associations exhibiting FDR‐significant *Q*_trait_ *P*‐values were not interpreted.

## 3 RESULTS

### 3.1 Genomic factor models

**FIGURE 1:**
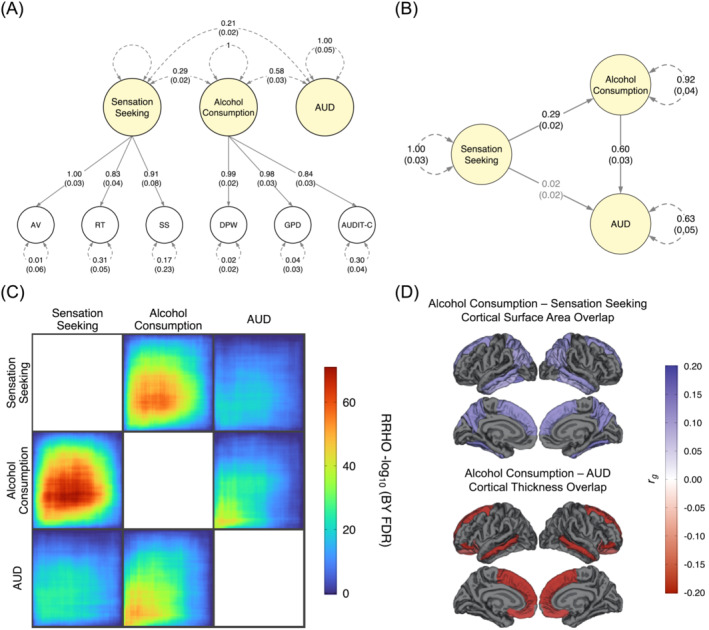
Examinations of shared and unique genetic architecture among sensation seeking, alcohol consumption, and AUD. (A) Path diagram of three‐factor model for sensation seeking, alcohol consumption, and AUD estimated in GenomicSEM. Presented parameters are standardised and *SEs* are shown in paratheses. Variances and covariances are shown as dashed lines, and factor loadings are shown as solid lines. See Table S4 for model fit indices. AV = adventurousness; RT = risk taking; SS = UPPS‐P sensation seeking; DPW = GSCAN drinks per week; GPD = UKB + AlcGen + CHARGE Plus grams of alcohol per day; AUDIT‐C = UKB + MVP AUDIT consumption score. (B) Path diagram of mediated path model between sensation seeking and AUD via alcohol consumption. Presented parameters are standardised and *SEs* are shown in paratheses. Grey coefficients are non‐significant (*P* = 0.324). Variances are shown as dashed lines, and regression paths are shown as solid lines. (C) Rank–rank hypergeometric tests of overlap (RRHO) between traits. Upper triangle: dlPFC neuronal (CN) H‐MAGMA‐based index of genetic overlap. Lower triangle: midbrain dopaminergic neuronal (DA) H‐MAGMA‐based index of genetic overlap. RRHO −log10 *P*‐values adjusted by the Benjamini and Yekutieli procedure (BY FDR). (D) Cortical patterning of unique genetic correlations between alcohol consumption and sensation seeking among regional cortical surface area neuroimaging phenotypes (top) and between alcohol consumption and AUD among regional cortical thickness neuroimaging phenotypes (bottom). Genetic correlation values are based on constrained models.

Univariate and bivariate LDSC estimates for all indicator GWAS are shown in Tables S2 and S3. The correlated three‐factor GenomicSEM model provided good fit to the genetic covariance matrices among sensation seeking, alcohol consumption, and AUD (*χ* ^2^ = 117.89, *df* = 12, *P* = 1.63 × 10^−19^, AIC = 149.89, CFI = 0.99, SRMR = 0.06; Figure 1A; Table S4). Alcohol consumption and AUD were strongly correlated in this model (*r*_*g*_ = 0.58, *SE* = 0.03, *P* = 3.67 × 10^−120^), whereas sensation seeking was moderately correlated with both alcohol consumption (*r*_*g*_ = 0.29, *SE* = 0.02, *P* = 4.50 × 10^−53^) and AUD (*r*_*g*_ = 0.21, *SE* = 0.02, *P* = 2.04 × 10^−19^). Constraining correlations with the sensation seeking factor to equality suggested sensation seeking was more strongly correlated with alcohol consumption than AUD (Δ*χ* ^2^ = 271.06, *df* = 1, *P*_diff_ = 6.65 × 10^−61^). In follow‐up sensation seeking and alcohol consumption single factor models, loadings were large and significant, and residual variances were generally small (Table S5).

### 3.2 Stratified models of heritability and genetic (co)variance

Twenty‐two, 46, and 24 functional annotations were significantly enriched for sensation seeking, alcohol consumption, and AUD, respectively, following FDR correction (Table S6). Across all three traits, significant enrichment was observed for gene expression in the frontal cortex; the H3K4me1 transcriptional enhancer in the dlPFC, inferior temporal lobe, and middle hippocampus; the H3K27ac promoter in the dlPFC and inferior temporal lobe; and the H3K9ac promoter in the inferior temporal lobe and anterior caudate. Unique enrichment of sensation seeking and alcohol consumption and their covariance, but not for AUD, was observed for H3K4me1 in the anterior caudate. Unique significant enrichment for sensation seeking and alcohol consumption, but not for their covariance or AUD, was also observed for H3K4me1 in the substantia nigra, H3K27ac in the cingulate cortex, and H3K9ac in the angular gyrus. No significant overlap in enrichment for sensation seeking and AUD was observed that did not also include alcohol consumption. However, there was common enrichment of gene expression in the anterior cingulate and cerebellum, H3K27ac in the anterior caudate, and the H3K4me3 transcriptional activator in the cingulate gyrus for alcohol consumption and AUD, which was not observed for sensation seeking.

### 3.3 Multivariate GWAS

**FIGURE 2:**
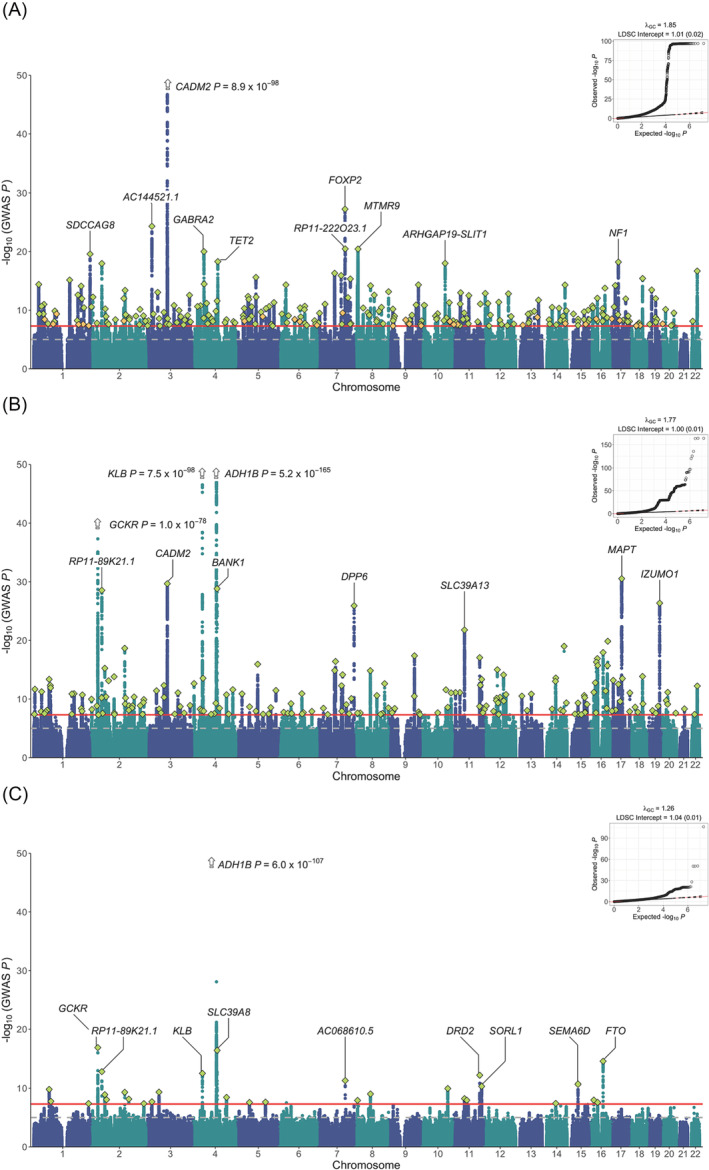
Multivariate genome‐wide association analysis of (A) sensation seeking, (B) alcohol consumption, and (C) AUD. Manhattan plot of –log10 (two‐sided) *P*‐value for GenomicSEM and METAL GWAS associations (main) and *Q*–*Q* plot of expected versus observed –log10 *P*‐values (upper right corners). Solid red line of Manhattan plots denotes genome‐wide significant (GWS) threshold (*P* < 5 × 10^−8^), and dashed grey line denotes *P* < 1 × 10^−5^. Mapped genes for top 10 associations are labelled. Green diamonds represent lead SNPs from independent GWS genomic loci. Orange diamonds (for sensation seeking; A) represent lead SNPs from novel GWS genomic loci green not previously reported in GWAS catalogue, Karlsson Linnér et al., 2021, or Sanchez‐Roige et al., 2023.

Manhattan and *Q*‐*Q* plots for sensation seeking, alcohol consumption, and AUD GWAS are shown in Figure 2. In the sensation seeking GWAS ($\hat{N}$ = 710,971; *ζ*_*g*_ = 0.087, *SE* = 0.003), 1092 independent GWS variants, mapped to 262 independent genomic loci, were identified (Tables S7 and S8). Thirty‐one genomic loci (~12%) represented novel loci that were not (A) associated with or in LD (*r* ^2^ > .1) with SNPs associated with any of the three sensation seeking indicator GWAS, (B) previously associated with any trait for studies included in the NHGRI‐EBI GWAS catalogue (version 104: 2021‐09‐15),^52^ and (C) not identified in other recently conducted trait‐relevant GWAS (e.g., impulsive personality traits^53^ and externalising behaviour^54^; Table S9). However, 14 of these loci map to genes previously associated with these traits through positional mapping of independent loci or gene‐based tests (Table 2). The top association in the sensation seeking GWAS was an intronic *CADM2* variant (rs2069123, *P* = 8.85 × 10^−98^) identified in prior GWAS of related traits.^21^, ^22^, ^53^, ^54^ The alcohol consumption GWAS ($\hat{N}$ = 1,388,120; *ζ*_*g*_ = 0.040, *SE* = 0.002) detected 842 independent GWS SNPs constituting 188 independent genomic loci (Tables S10 and S11). The top hit in this GWAS was an intergenic variant (rs138495951, *P* = 5.20 × 10^−165^) mapped to the alcohol metabolism gene *ADH1B*, replicating previous GWAS for alcohol traits.^19^, ^23^, ^28^ The AUD GWAS meta‐analysis (*n*_effective_ = 220,182; $h_{g}^{2}$ = 0.086, *SE* = 0.004; liability‐scale $h_{g}^{2}$ = 0.237, *SE* = 0.012) detected 65 independent GWS SNPs constituting 31 independent genomic loci (Tables S12 and 13). The top hit in this GWAS was also a common *ADH1B* variant (rs1229984, *P* = 5.99 × 10^−107^), again replicating prior studies.^27^, ^28^ Univariate LDSC results for all three GWAS and *Q*_SNP_ summaries for sensation seeking and alcohol consumption are reported in Table S14 and bivariate LDSC results in Table S15.

**TABLE 2:** Lead variants for 31 independent novel genome‐wide significant loci (*P* < 5 × 10^−8^) for sensation seeking factor.

rsID	Chr	Position	Gene	A1	A2	MAF	*Z*	*P*	sigMAGMA	sigH‐MAGMA dlPFC	sigH‐MAGMA DA‐midbrain
rs62487983	7	103877127	*ORC5* ^+^	C	T	0.23	6.30	2.95 × 10^−10^			
rs17117562	1	98564736	*NFU1P2*	T	C	0.36	−6.24	4.39 × 10^−10^			
rs2925091	5	109385,165	*PGAM5P1*	G	A	0.23	6.07	1.25 × 10^−9^			
rs7337720	13	94205582	*GPC6* ^+^	G	A	0.40	−6.03	1.61 × 10^−9^	*		
rs8066604	17	1237482	*YWHAE*	C	T	0.42	5.93	3.02 × 10^−9^	*		
rs9922316	16	23909952	*PRKCB* ^+^	T	G	0.19	−5.92	3.13 × 10^−9^	*		
rs74486058	1	46787455	*UQCRH* ^+^	A	G	0.14	−5.90	3.74 × 10^−9^	*		
rs80137000	6	67393316	*RP11‐24C14.1* ^+^	G	A	0.06	−5.89	3.75 × 10^−9^			
rs10869253	9	71346430	*PIP5K1B*	C	T	0.48	−5.86	4.65 × 10^−9^		*	
rs11653093	17	35547802	*ACACA*	C	T	0.48	5.83	5.55 × 10^−9^	*		
rs1405929	2	140842377	*AC073928.2*	G	A	0.45	5.80	6.74 × 10^−9^			
rs7704530	5	30845465	*RPL19P11* ^+^	G	A	0.30	−5.78	7.37 × 10^−9^			
rs6585468	10	119630558	*RP11‐355F22.1*	G	A	0.35	−5.76	8.40 × 10^−9^			
rs10944412	6	89592350	*RNGTT*	C	T	0.28	5.75	8.87 × 10^−9^			
rs58948914	14	99536354	*AL162151.4* ^+^	C	T	0.05	5.62	1.88 × 10^−8^			
rs3810561	20	3211064	*SLC4A11*	A	C	0.29	−5.60	2.18 × 10^−8^			
rs1332893	9	87250808	*RP11‐361M4.1*	C	G	0.09	−5.59	2.25 × 10^−8^			
rs10874390	1	84004080	*RP11‐475O6.1*	C	T	0.42	−5.59	2.32 × 10^−8^			
rs4384208	1	213950251	*RP11‐323I1.1*	T	G	0.15	5.58	2.45 × 10^−8^			
rs7167767	15	81025755	*ABHD17C* ^+^	G	A	0.33	−5.57	2.49 × 10^−8^	*		
rs4757587	11	17760904	*KCNC1*	C	T	0.16	5.56	2.64 × 10^−8^			
rs7543251	1	193820890	*RPL23AP22*	A	G	0.16	−5.56	2.67 × 10^−8^			
rs61869029	11	1472204	*BRSK2* ^+^	G	A	0.20	5.56	2.74 × 10^−8^			*
rs9578192	13	31254866	*USPL1*	A	G	0.45	−5.51	3.61 × 10^−8^			
rs1764022	6	163809600	*CAHM* ^+^	G	A	0.07	−5.50	3.72 × 10^−8^			
rs9944845	18	42487993	*SETBP1* ^+^	A	G	0.40	−5.49	4.12 × 10^−8^	*	*	*
rs16835705	1	237908911	*RYR2* ^+^	G	A	0.28	5.47	4.49 × 10^−8^	*		
rs4239453	18	25413480	*CDH2*	C	T	0.26	−5.47	4.49 × 10^−8^			*
rs7869395	9	137976605	*OLFM1* ^+^	G	A	0.19	5.47	4.53 × 10^−8^	*		
rs17678776	18	26170337	*ARIH2P1*	T	A	0.14	−5.46	4.70 × 10^−8^			
rs6475044	9	16355870	*BNC2* ^+^	C	T	0.49	5.45	4.91 × 10^−8^	*		*

*Note*: Chr is the chromosome number of SNP; Position is the base position of SNP on hg19; A1 is the effect allele based on ANNOVAR annotations; A2 is reference allele; MAF is minor allele frequency in 1000 Genomes; sigMAGMA, sigH‐MAGMA dlPFC, and sigH‐MAGMA DA‐midbrain are genes that were associated in the MAGMA and the dlPFC neuron and midbrain dopaminergic neuron H‐MAGMA analyses, respectively. A ‘+’ following the gene name indicates that while the reported locus was novel, prior studies of externalising behaviour and/or sensation seeking traits^21^, ^22^, ^53^, ^54^ had implicated the gene through associations with independent loci or gene‐based tests (MAGMA, H‐MAGMA, S‐PrediXcan, and/or DEPICT).

### 3.4 Post‐multivariate GWAS analyses

#### 3.4.1 Mediation model

The mediation analysis suggested a negligible direct association between sensation seeking and AUD (*β*_*g*_ = 0.02, *SE* = 0.02, *P* = .324) after accounting for an alcohol consumption‐mediated pathway (Figure 1B; Table S16). The significance of the indirect path, calculated using the Sobel test^55^ (*β*_*g*_ = 0.17, *SE* = 0.01, *P* = 2.73 × 10^−39^), suggested full mediation.

#### 3.4.2 Gene‐based analyses

MAGMA identified 452 genes associated with sensation seeking, 483 with alcohol consumption, and 30 with AUD. H‐MAGMA identified 476 dlPFC and 686 DA‐midbrain tissue‐cell‐type‐specific genes for sensation seeking (Tables S17–S19), 548 dlPFC and 726 DA‐midbrain genes for alcohol consumption (Tables S20–22), and 43 dlPFC and 52 DA‐midbrain genes for AUD (Tables S23–25). When overlapping MAGMA and H‐MAGMA results were restricted to genes that also demonstrated evidence of association via positional mapping of GWS loci, 46 and 43 genes showed enrichment with sensation seeking and alcohol consumption, respectively, across both H‐MAGMA tissue‐cell types (i.e., DA‐midbrain and dlPFC neurons). Three genes (*ADH1B, DRD2*, and *RHOA*) showed similar enrichment across tissue‐cell types for AUD. For tissue‐specific results, sensation seeking and alcohol consumption demonstrated greater H‐MAGMA evidence for enrichment in DA‐midbrain (23 and 17 genes, respectively) relative to dlPFC neurons (10 and 7 genes, respectively). For AUD, only a single gene identified by both positional mapping and MAGMA showed H‐MAGMA tissue‐specific enrichment: *PDE4B* in dlPFC neurons. When looking for overlap across traits (i.e., genes mapped positionally and by MAGMA but to just one H‐MAGMA annotation in relation to two or more traits), only two genes were identified: *SEMA6D* and *RUNX1T1* showed enrichment in DA‐midbrain neurons for both sensation seeking and alcohol consumption.

Pairwise RRHO analyses of H‐MAGMA gene enrichment indicated that overlap of pleiotropic genes between traits was stronger for DA‐midbrain than for dlPFC neurons, particularly for sensation seeking and alcohol consumption, where the number of pleiotropic genes was ~2 times higher in DA‐midbrain than in dlPFC neurons. Visual comparisons of results from pairwise RRHO outputs using heatmaps (Figure 1C) also suggested that the overlap between sensation seeking and alcohol consumption was stronger (*Z*_CN_ = 16.11; *Z*_DA_ = 17.85) than the overlap between sensation seeking and AUD (*Z*_CN_ = 9.47; *Z*_DA_ = 10.53) and alcohol consumption and AUD (*Z*_CN_ = 14.20; *Z*_DA_ = 15.25). This was particularly true for DA‐midbrain neurons where the number of overlapping genes between sensation seeking and alcohol consumption was ~2.5 and ~4.5 times higher than that between sensation seeking and AUD and alcohol consumption and AUD, respectively.

#### 3.4.3 Local genetic correlation analyses

In total, 47 significant bivariate local genetic correlations were identified among sensation seeking, alcohol consumption, and AUD, following Bonferroni correction (Table S26). Sensation seeking and alcohol consumption were positively correlated across 26 loci (mean *ρ*_*g*_ = 0.71). At 14 of these loci, the 95% confidence intervals for the explained variance included 1, suggesting complete overlap of genetic signals. In contrast, sensation seeking was only correlated with AUD across four loci (three positive: *ρ*_*g*_ = 0.39–0.46; one negative: *ρ*_*g*_ = −0.56). Alcohol consumption and AUD were correlated across 17 loci (16 positive: *ρ*_*g*_ = 0.36–0.75; one negative: *ρ*_*g*_ = −0.53). None of these confidence intervals of explained variance included 1.

Significant local genetic correlations involving all three phenotypes were observed at two loci. At the first locus (chr4:102,544,804‐104,384,534 containing the *BANK1* and *SLC39A8* genes; alcohol consumption‐sensation seeking‐*ρ*_*g*_ = 0.57, *P* = 1.90 × 10^−5^; alcohol consumption‐AUD‐*ρ*_*g*_ = 0.46, *P* = 1.70 × 10^−8^), follow‐up partial correlation tests, conditioning bivariate correlations on the third phenotype (e.g., sensation seeking‐alcohol consumption‐*ρ*_*g*_ partialling out AUD) demonstrated only small changes in association estimates, and a multiple regression model with sensation seeking and AUD predicting alcohol consumption demonstrated a substantial increase in variance explained. These results suggest alcohol consumption is uniquely related to sensation seeking and AUD at this locus (Table 3A). Conversely, at the second locus (chr9:128,785,784‐129,617,771 containing the *MVB12B* and *LMX1B* genes; sensation seeking‐alcohol consumption‐*ρ*_*g*_ = 0.75, *P* = 1.13 × 10^−10^; sensation seeking‐AUD‐*ρ*_*g*_ = 0.39, *P* = 2.53 × 10^−7^), partial correlation and multiple regression tests demonstrated overlapping associations with sensation seeking, such that alcohol consumption, rather than AUD, was largely responsible for the variance explained in sensation seeking at this locus (Table 3B).

**TABLE 3:** Conditional local genetic relations among sensation seeking, alcohol consumption, and AUD.

A. Conditional local genetic associations for alcohol consumption within the *BANK1‐SLC39A8* gene locus (chr4:102,544,804‐104,384,534)				
Model	Phenotype	Parameter	*R* ^2^	*P*
Bivariate	SS	0.57	0.21	**1.90 × 10** ^**−5**^
Bivariate	AUD	0.46	0.32	**1.70 × 10** ^**−8**^
Partial correlation	SS| AUD	0.61		3.87 × 10^−5^
Partial correlation	AUD| SS	0.51		4.17 × 10^−4^
Multiple regression	SS	0.54	0.50	2.71 × 10^−4^
	AUD	0.42		1.26 × 10^−3^

B. Conditional local genetic associations for sensation seeking within the *MVB12B‐LMX1B* gene locus (chr9:128,785,784‐129,617,771)				
Model	Phenotype	Parameter	*R* ^2^	*P*
Bivariate	ALC	0.75	0.56	**1.13 × 10** ^**−10**^
Bivariate	AUD	0.39	0.15	**2.53 × 10** ^**−7**^
Partial correlation	ALC| AUD	0.72		**3.50 × 10** ^**−8**^
Partial correlation	AUD| ALC	0.27		1.08 × 10^−1^
Multiple regression	ALC	0.69	0.59^a^	**2.01 × 10** ^**−5**^
	AUD	0.21		1.17 × 10^−1^

*Note*: For bivariate/partial correlation models, parameter = *ρ*_*g*_. For multiple regression, parameter = local *β*_*g*_. *P*‐values shown in **bold** are Bonferroni significant.

Abbreviations: ALC = alcohol consumption; AUD = alcohol use disorder; SS = sensation seeking.

^a^ 95% CI includes 1.

#### 3.4.4 Neuroimaging genetic correlation analyses

Associations presented below were FDR significant without evidence of *Q*_trait_ heterogeneity. For regional cortical volume, genetic correlation estimates yielded a single FDR‐significant association between AUD and the left pars orbitalis (*r*_*g*_ = 0.19; Table S27). For subcortical structure volume, there were no FDR‐significant associations with any of the traits (Table S28). For cortical surface area, nine regions were significantly positively correlated with sensation seeking (*r*_*g*_ = 0.08–0.10) and 21 regions were significantly positively correlated with alcohol consumption (*r*_*g*_ = 0.08–0.18; Table S29). Seven overlapping cortical surface area associations, primarily localised to temporal and parietal areas in addition to the superior frontal gyrus, did not significantly differ in their magnitude between sensation seeking and alcohol consumption (Figure 1D). For cortical thickness, considerable overlap across regions was observed between alcohol consumption (26 regions: *r*_*g*_ = −0.07 to −0.17) and AUD (18 regions: *r*_*g*_ = −0.11 to −0.20; Table S30), and overlapping associations that did not significantly differ in magnitude were localised to four frontal (lateral/medial orbitofrontal, pars orbitalis, and superior frontal) and the middle temporal regions (Figure 1D).

For rs‐fMRI connectivity phenotypes, there were no FDR‐significant genetic correlations with alcohol consumption or AUD (Table S31). However, sensation seeking was significantly correlated with 17 rs‐fMRI functional connectivity phenotypes, including a global connectivity measure broadly indexing functional connectivity between motor and subcortical‐cerebellar networks (*r*_*g*_ = 0.25). To simplify the examination and interpretation of single network measure results, connectivity nodes were manually mapped to a 7‐network parcellation^56^: frontoparietal, visual, limbic, dorsal attention, ventral attention, somatomotor, and default networks. Based on this parcellation, results suggested consistent genetic overlap between sensation seeking and connectivity phenotypes involving somatomotor networks as well as nodes localised to the cerebellum (Table 4).

**TABLE 4:** FDR‐significant genetic correlations between sensation seeking and 16 rs‐fMRI network connectivity phenotypes.

Node 1		Node 2		*r*_*g*_	*SE*	*P*_FDR_
Network	Primary localisation	Network	Primary localisation			
Somatomotor	Paracentral lobule	Somatomotor	Pre/postcentral gyrus	0.19	0.07	2.70 × 10^−2^
Somatomotor	Postcentral gyrus	Somatomotor	Paracentral lobule	0.24	0.06	4.66 × 10^−4^
Somatomotor	Post/precentral gyrus	Somatomotor	Postcentral gyrus	0.16	0.04	2.73 × 10^−3^
Cerebellum	Cerebellum	Somatomotor	Post/precentral gyrus	−0.22	0.07	9.87 × 10^−3^
Cerebellum	Cerebellum/cerebellum crus	Somatomotor	Paracentral lobule	−0.25	0.05	1.28 × 10^−5^
Cerebellum	Cerebellum/cerebellum crus	Somatomotor	Post/precentral gyrus	−0.24	0.06	8.03 × 10^−4^
Cerebellum	Cerebellum	Somatomotor	Post/precentral gyrus	−0.26	0.08	8.18 × 10^−3^
Cerebellum	Cerebellum	Somatomotor	Paracentral lobule	−0.19	0.05	2.60 × 10^−3^
Cerebellum	Cerebellum/cerebellum crus	Limbic	Putamen	0.31	0.07	4.66 × 10^−4^
Somatomotor	Paracentral lobule	Dorsal attention	Post/precentral gyrus	0.27	0.06	4.66 × 10^−4^
Somatomotor	Postcentral gyrus	Dorsal attention	Post/precentral gyrus	0.23	0.07	3.60 × 10^−3^
Somatomotor	Paracentral lobule	Default	Superior temporal gyrus	0.21	0.07	2.51 × 10^−2^
Somatomotor	Postcentral gyrus	Limbic	Putamen	−0.29	0.09	8.87 × 10^−3^
Somatomotor	Post/precentral gyrus	Limbic	Putamen	−0.21	0.06	2.82 × 10^−3^
Dorsal attention	Precentral/frontal superior gyrus	Ventral attention	Rolandic Operculum/supramarginal gyrus	−0.21	0.05	4.66 × 10^−4^
Dorsal attention	Post/precentral gyrus	Limbic	Putamen	−0.31	0.09	3.37 × 10^−3^

*Note*: All correlations differed significantly in magnitude compared to alcohol consumption and AUD. Functional brain regions (ROIs) manually mapped to networks based on seven‐network parcellation.^56^ Primary localisation defined by top‐two tagged automated anatomical labelling (AAL) regions ranked by the number of voxels overlapped with an ROI. *r*_*g*_ reflects genetic correlations between sensation seeking and co‐activity between nodes 1 and 2, that is, positive correlations demonstrate genetic overlap between higher sensation seeking and enhanced functional connectivity, and negative correlations demonstrate genetic overlap between higher sensation seeking and reduced functional connectivity.

## 4 DISCUSSION

Previous research has demonstrated important phenotypic, neurobiological, and genetic overlap among sensation seeking, alcohol consumption, and AUD. This research has informed current addictions theory and suggests that sensation seeking, especially in adolescence and emerging adulthood, functions as a significant risk factor for heavy alcohol consumption, which may subsequently lead to AUD development.^2^, ^3^ The current study's extensive examination of these traits and their overlap resulted in five main findings: (1) replication of associated loci for alcohol consumption and AUD; (2) identification of novel GWS loci for sensation seeking; (3) stronger genetic overlap between sensation seeking and alcohol consumption than between sensation seeking and AUD with alcohol consumption mediating the genetic relation between sensation seeking and AUD; (4) genetic overlap between sensation seeking and alcohol consumption was related to neural regions/circuits central to neurobiological models of addiction (e.g., midbrain dopaminergic neurons and anterior caudate); and (5) genetic overlap between alcohol consumption and AUD was characterised by decreased frontocortical thickness and by genetic variation in a gene region (*BANK1*‐*SLC39A8*) previously implicated in striatal volume.^26^, ^30^ These are discussed in turn.

First, variant‐level results for the reported alcohol consumption and AUD GWAS largely replicated previous findings (e.g., variation in *ADH1B*).^19^, ^23^, ^27^, ^28^ Results of additional gene‐level and neuroimaging genetic correlation analyses also corroborate results from prior studies. For AUD, gene‐based associations with the D2 dopamine receptor gene (*DRD2*) were observed along with dlPFC neuronal tissue‐specific enrichment of *PDE4B*, which encodes for an enzyme involved in dopamine signalling, highlighting regulatory effects on dopaminergic pathways recently linked to a broad genetic addiction liability factor.^57^ Further, the association between AUD and variants related to decreased volume of the left pars orbitalis replicates recent Mendelian randomisation findings examining causal influences of brain morphology on problematic alcohol use.^58^

Second, the sensation seeking GWAS identified variants and genes (e.g., *CADM2*, *NF1*, *FOXP2*, *GABRA2*) previously associated with indicator traits and related substance use and self‐regulatory traits.^22^, ^23^, ^54^, ^59^ Further, 31 genomic loci representing new discoveries in the documented literature were identified, 17 of which mapped to genes not previously implicated in sensation seeking or externalising behaviour GWAS, highlighting the benefit of increased power afforded by multivariate GWAS approaches for variant discovery. Gene‐based analyses suggested that positionally mapped genes for several of these novel loci have important neurobiological functions. For example, rs9944845, mapped to an intronic region of *SETBP1*, was associated with sensation seeking and contributed to significant associations across all gene‐based analyses. *SETBP1* encodes a DNA‐binding protein involved in activating gene expression through recruitment of epigenomic protein complexes and has been linked to neurogenesis and neuronal migration.^60^ A prior study also reported associations between an intergenic locus near *SETBP1* and externalising behaviour that was further substantiated by gene‐based tests providing additional support for the novel result reported here.^54^ Other genes showed more specific tissue and cell‐type associations. For example, *BRSK2* (rs61869029), which encodes a protein involved in neuron polarisation and axonogenesis and has been previously implicated in risk taking,^22^ and *CDH2* (rs4239453), which encodes a protein involved in cell‐to‐cell adhesion, both showed DA‐midbrain specificity in H‐MAGMA analyses.

Building on these results, sensation seeking demonstrated genetic correlations with resting‐state functional connectivity phenotypes. Specifically, sensation seeking was associated with increased somatomotor intra‐connectivity and decreased connectivity between the somatomotor and cerebellar networks, which are increasingly emphasised in neurobiological models of addiction.^61^ Sensation seeking was also consistently negatively correlated with functional connectivity between somatomotor/dorsal attention networks and the putamen, highlighting shared genetic influences between sensation seeking and reduced co‐activity along parieto‐limbic pathways.^62^ In aggregate, the sensation seeking GWAS and follow‐up analyses provide evidence of genetic influences implicating plausible neurobiological mechanisms involved in the aetiology of sensation seeking (e.g., neuronal development, cell signalling, and dopaminergic/reward pathways) that extend beyond the hypothesised subcortical regions associated with ‘bottom‐up’ processes.

Third, GenomicSEM analyses provided novel evidence suggesting greater genome‐wide genetic overlap between sensation seeking and alcohol consumption (*r*_*g*_ = 0.29) than between sensation seeking and AUD (*r*_*g*_ = 0.21). Although the magnitude of this difference was relatively small, mediation analyses demonstrated that after accounting for the genetic association with alcohol consumption, sensation seeking was not associated with AUD. These findings were consistent with local genetic correlation analyses highlighting both partial and complete overlap of genetic signal between sensation seeking and alcohol consumption at a much greater number of loci relative to AUD. Moreover, at the *MVB12B*‐*LMX1B* locus, which includes genes with critical roles in protein sorting with enriched expression in brain tissues (*MVB12B*)^39^ and midbrain dopaminergic neuronal differentiation and survival (*LMX1B*),^63^ the observed correlation between sensation seeking and AUD was largely accounted for by the relation of alcohol consumption with sensation seeking. These results demonstrate that genetic influences underlying associations between sensation seeking and AUD are largely mediated by increased alcohol consumption, consistent with prior neurobiological models relevant to addiction (e.g., dual‐systems and ‘addiction cycle’ models^8^, ^11^) and phenotypic research emphasising this pathway in early drinking experiences.^2^, ^3^, ^8^

Fourth, post‐GWAS follow‐up analyses examining neurogenetic and multi‐omic overlap between sensation seeking and alcohol consumption identified associations with genes implicating midbrain dopaminergic neurons (H‐MAGMA) and transcriptional enhancement in the dorsal striatum (stratified GenomicSEM), highlighting key reward pathways associated with heavy alcohol consumption and AUD. The shared enrichment in regions implicated in addiction neurobiology emphasises the critical role of ascending dopaminergic pathways from the basal ganglia and midbrain regions mediating the link between reward‐based incentives and alcohol consumption.^11^, ^12^ This finding partially replicates recent associations from similar H‐MAGMA analyses^64^ and quantitative susceptibility mapping,^65^ collectively suggesting that a component of genetic risk for increased alcohol consumption localises to midbrain and basal ganglia structures, and provides an important extension by suggesting that increased sensation seeking may partially explain these previously observed gene–brain–behaviour relations.

In addition to subcortical neurogenetic findings, sensation seeking and alcohol consumption exhibited overlapping genetic associations with cortical surface area in frontal, temporal, and parietal regions. These findings support prior studies emphasising associations between sensation seeking and increased frontotemporal surface area in adolescents,^13^, ^66^ and shared genetic influences between alcohol consumption and greater total cortical surface area.^67^ Interestingly, H‐MAGMA dopaminergic midbrain‐specific gene associations for sensation seeking (*BRSK2*) and both sensation seeking and alcohol consumption (*RUNX1T1*) highlight genes previously implicated in surface area and sulcal depth.^68^, ^69^ Thus, these shared associations between sensation seeking and alcohol consumption point to genes that may have pleiotropic influences on both midbrain dopaminergic neurons and cortical surface area, providing a potential explanation for observed associations between sensation seeking and cortical neuroimaging phenotypes that would be hypothesised as more relevant to ‘top‐down’ cognitive control processes.^8^, ^18^

Fifth, important sources of overlap between alcohol consumption and AUD (*r*_*g*_ = 0.58) were observed, adding to the literature examining their genetic commonalities and differences.^19^, ^20^, ^28^ One source of overlap was the *BANK1*‐*SLC39A8* locus, a region of relatively high LD that includes two genes ~175 kb apart that code for proteins involved in cellular transport and scaffolding. Multiple analytic approaches (e.g., GWAS, LAVA, and H‐MAGMA) in the present study linked this region to alcohol consumption and AUD, whereas prior studies reported only suggestive associations with alcohol consumption traits.^70^ Notably, H‐MAGMA analyses indicated tissue‐specific relations to gene annotations in dopaminergic midbrain neurons, consistent with prior evidence indicating that variants in this region are associated with striatal volume,^26^, ^30^, ^71^ and act as *cis*‐expression quantitative trait loci for these genes in the caudate and putamen.^39^ Taken together, these results highlight a shared *BANK1*‐*SLC39A8* genetic signal relevant to the mesostriatal dopaminergic pathway implicated in neurobiological models of addiction.^11^

Another observed source of overlap between alcohol consumption and AUD was in shared genetic correlations with decreased cortical thickness that was particularly pronounced in frontal regions. To date, research examining genetic correlations between alcohol use traits and neuroimaging phenotypes using large‐scale GWAS data is relatively limited.^58^, ^67^ Given that reduced frontocortical thickness has previously been associated with impulsive choice in adolescence,^72^ alcohol consumption in young adults,^73^ and AUD in middle‐aged adults,^74^ the present results highlight a possible genetic pathway consistent with addiction theory whereby genetic liability for reduced cortical thickness leads to disinhibited behaviour, which may confer greater risk for transitions from heavy consumption to AUD. Future studies using longitudinal data to test this pathway more directly are needed to fully elucidate the neurogenetic features influencing progression to AUD.

In sum, these analyses underscore the importance of using neurobiologically informed annotation datasets and neuroimaging data to further elucidate biological substrates underlying genetic overlap between traits characterising AUD development. While the current study is informative, it is not without limitations. First, analyses were restricted to samples of European ancestry and cannot be generalised to other ancestral populations, which are greatly underrepresented in GWAS research.^75^ Current initiatives to extend GWAS of alcohol use traits across ancestral populations may help address this limitation in future research.^76^ Second, the sample size of the AUD GWAS, though one of the larger GWAS of AUD diagnosis to date, was less well‐powered relative to sensation seeking and alcohol consumption. Sample size discrepancies across traits and related differences in power to detect associations may have influenced findings to some extent.

## 5 CONCLUSION

Genetic and neurogenetic variant‐level, gene‐level, and tissue‐specific analyses of sensation seeking, alcohol consumption, and AUD demonstrated both common and unique associations across these traits emphasising specific neurobiological pathways. Phenotypic correlations between sensation seeking and alcohol consumption, and alcohol consumption and AUD, though both influenced, in part, by genes implicated across mesocorticolimbic reward circuitry, may also reflect separable genetic architectures with unique neurobiological substrates. Taken together, observed associations support a biologically plausible, genetically mediated pathway from sensation seeking to heavy alcohol consumption to AUD development characterised by a progressive pattern of use consistent with neurobiological models of addiction.

## ETHICS APPROVAL STATEMENT

All secondary data analysis of GWAS summary statistics and reference panels were considered exempt by the Institutional Review Board at the University of Missouri.

## AUTHOR CONTRIBUTION

Alex P. Miller and Ian R. Gizer were responsible for the study concept and design, data acquisition, interpretation of findings, and drafted and revised the manuscript. Alex P. Miller performed data analysis. All authors critically reviewed content and approved the final version for publication.

## CONFLICT OF INTEREST STATEMENT

The authors declare none.

## Supporting information


**Figure S1.** **Q‐Q plots for GenomicSEM indicator GWAS meta‐analyses** *.* These results have not been adjusted for genomic control inflation factors (λ_GC_). **(A)** 23andMe + Linnér et al risk taking meta‐analysis. **(B)** UK Biobank + Million Veteran Program AUDIT‐C meta‐analysis. **(C)** 23andMe + GWAS & Sequencing Consortium of Alcohol and Nicotine use (GSCAN) drinks per week meta‐analysis.
**Figure S2** *. **Q***_**SNP**_ **analysis of (A) sensation seeking and (B) alcohol consumption.** Manhattan plot of –log10 (two‐sided *Q*_SNP_ *P*‐value) for GenomicSEM associations (main) and Q‐Q plot of expected vs. observed –log10 *Q*_SNP_ *P*‐values (upper right corners). Solid red line of Manhattan plots denotes genome‐wide significant (GWS) threshold (*P* < 5 × 10^−8^) and dashed grey line denotes *P* < 1 × 10^−5^. Purple diamonds represent GWS *Q*_SNPs_ (*n* = 21 for alcohol consumption).

**Data S1.** Supporting Information.

**Table S1.** LDSC genetic correlations between meta‐analysed samples.
Table S2.Univariate LDSC estimates of indicator phenotypes included in GenomicSEM models.
Table S3. Bivariate LDSC estimates of indicator phenotypes included in GenomicSEM models.
Table S4. Confirmatory analysis of two‐ and three‐factor models of sensation seeking, alcohol consumption, and AUD.
Table S5. Confirmatory analysis of single factor models.
Table S6. Genetic enrichment of sensation seeking, alcohol consumption, and AUD factors estimated using stratified GenomicSEM and LDSC‐SEG.
Table S7. 1,092 independent GWS SNPs (*P* < 5 × 10^−8^) for sensation seeking factor ordered by chromosomal location.
Table S8. 262 independent GWS loci (*P* < 5 × 10^−8^) for sensation seeking factor ordered by chromosomal location.
Table S9. Lookup of the 1,092 independent GWS sensation seeking factor SNPs and correlated SNPs (*r* ^2^ > 0.1) in the GWAS Catalogue, Línner et al, 2021, and Sanchez‐Roige et al, 2022.
Table S10. 842 independent GWS SNPs (*P* < 5 × 10^−8^) for alcohol consumption factor ordered by chromosomal location.
Table S11. 188 independent GWS loci (*P* < 5 × 10^−8^) for alcohol consumption factor ordered by chromosomal location.
Table S12. 65 independent GWS SNPs (*P* < 5 × 10^−8^) for AUD meta‐analysis ordered by chromosomal location.
Table S13. 31 independent GWS loci (*P* < 5 × 10^−8^) for AUD meta‐analysis ordered by chromosomal location.
Table S14. Univariate LDSC estimates of factor phenotypes derived from GenomicSEM and METAL.
Table S15.Bivariate LDSC estimates of factor phenotypes derived from GenomicSEM and METAL.
Table S16. Confirmatory model of mediated path between sensation seeking and AUD via alcohol consumption.
Table S17. 452 Bonferroni significant results (*P* < 2.66 × 10^−6^) of gene‐based association analyses (MAGMA) for sensation seeking factor.
Table S18. 476 Bonferroni significant results (*P* < 2.67 × 10^−6^) of gene based association analyses (H‐MAGMA, dlPFC cortical neurons) for sensation seeking factor.
Table S19. 686 Bonferroni significant results (*P* < 2.68 × 10^−6^) of gene based association analyses (H‐MAGMA, dopaminergic midbrain neurons) for sensation seeking factor.
Table S20. 483 Bonferroni significant results (*P* < 2.66 × 10^−6^) of gene‐based association analyses (MAGMA) for alcohol consumption factor.
Table S21. 548 Bonferroni significant results (*P* < 2.67 × 10^−6^) of gene based association analyses (H‐MAGMA, dlPFC cortical neurons) for alcohol consumption factor.
Table S22. 726 Bonferroni significant results (*P* < 2.68 × 10^−6^) of gene based association analyses (H‐MAGMA, dopaminergic midbrain neurons) for alcohol consumption factor.
Table S23. 30 Bonferroni significant results (*P* < 2.63 × 10^−6^) of gene‐based association analyses (MAGMA) for AUD meta‐analysis.
Table S24. 43 Bonferroni significant results (*P* < 2.66 × 10^−6^) of gene based association analyses (H‐MAGMA, dlPFC cortical neurons) for AUD meta‐analysis.
Table S25. 52 Bonferroni significant results (*P* < 2.67 × 10^−6^) of gene based association analyses (H‐MAGMA, dopaminergic midbrain neurons) for AUD meta‐analysis.
Table S26. 47 LAVA Bonferroni‐significant results (*P* < 3.11 × 10^−5^) for local genetic correlations among sensation seeking, alcohol consumption, and AUD.
Table S27. Genetic correlations between sensation seeking, alcohol consumption, and AUD factors and regional cortical volume.
Table S28. Genetic correlations between sensation seeking, alcohol consumption, and AUD factors and subcortical structure volume.
Table S29. Genetic correlations between sensation seeking, alcohol consumption, and AUD factors and regional cortical surface area.
Table S30. Genetic correlations between sensation seeking, alcohol consumption, and AUD factors and regional cortical thickness.
Table S31. Genetic correlations between sensation seeking, alcohol consumption, and AUD factors and rs‐fMRI functional connectivity traits.

## Data Availability

The full GWAS summary statistics for the 23andMe discovery data set will be made available through 23andMe to qualified researchers under an agreement with 23andMe that protects the privacy of the 23andMe participants. Please visit https://research.23andme.com/collaborate/#dataset‐access/ for more information and to apply to access the data. GWAS summary statistics for risk taking in the UKB cohort along with 10 smaller replication samples were obtained from https://thessgac.com/. GWAS summary statistics for drinks per week were obtained from https://conservancy.umn.edu/handle/11299/201564. Meta‐analytic GWAS summary statistics (AlcGen, CHARGE +, and UKB) for grams of alcohol consumed per day were obtained through author request and the European Molecular Biology Laboratory's European Bioinformatics Institute website (http://ftp.ebi.ac.uk/). PGC alcohol dependence and UKB GWAS summary statistics for AUDIT‐C were obtained from the PGC website (https://www.med.unc.edu/pgc/). Million Veteran Program GWAS summary statistics were obtained through the Database for Genotypes and Phenotypes (dbGaP; Study Accession: phs001672). FinnGenR6 ICD‐based AUD GWAS data were obtained from https://r6.finngen.fi/pheno/AUD. For more information, visit https://finngen.gitbook.io/documentation/. GWAS summary statistics from UKB cortical regional volume neuroimaging phenotypes were obtained using the Oxford Brain Imaging Genetics (BIG40) web server (https://open.win.ox.ac.uk/ukbiobank/big40/). ENIGMA and UKB GWAS summary statistics for cortical thickness and surface area and CHARGE, ENIGMA, and UKB GWAS summary statistics for subcortical structural volume were obtained by request at http://enigma.ini.usc.edu/. GWAS summary statistics from UKB rs‐fMRI phenotypes were obtained using the Brain Imaging Genetics Knowledge Portal (BIG‐KP; https://bigkp.org). GTEx v8 RNA‐seq read counts and transcripts per million normalised gene expression data were obtained from https://gtexportal.org/home/datasets. The 1000 Genomes Project Phase 3 BaselineLD v2.2 and Roadmap Epigenomics Consortium annotation datasets for LDSC‐SEG and stratified GenomicSEM were obtained from https://alkesgroup.broadinstitute.org/LDSCORE/. Hi‐C datasets were obtained from the Won Lab GitHub repository (https://github.com/thewonlab/H-MAGMA). The locus file used for LAVA analyses was accessed at https://github.com/josefin-werme/LAVA/tree/main/support_data. The 1000 Genomes Project Phase 3 LD reference panel data for LDSC‐SEG, MAGMA and GenomicSEM/stratified GenomicSEM were obtained from https://alkesgroup.broadinstitute.org/LDSCORE/, https://ctg.cncr.nl/software/magma, and https://github.com/GenomicSEM/GenomicSEM, respectively.
