# Supplementary material for: Neurogenetic and multi‐omic sources of overlap among sensation seeking, alcohol consumption, and alcohol use disorder
Source: Addict Biol. 2024 Jan 29;29(2):e13365. doi: 10.1111/adb.13365 (PMC10882188; doi:10.1111/adb.13365)
Supplement: Supplementary file 1 — Figure S1. Q‐Q plots for GenomicSEM indicator GWAS meta‐analyses . These results have not been adjusted for genomic control inflation factors (λGC). (A) 23andMe + Linnér et al risk taking meta‐analysis. (B) UK Biobank + Million Veteran Program AUDIT‐C meta‐analysis. (C) 23andMe + GWAS & Sequencing Consortium of Alcohol and Nicotine use (GSCAN) drinks per week meta‐analysis. Figure S2 . Q SNP analysis of (A) sensation seeking and (B) alcohol consumption. Manhattan plot of –log10 (two‐sided Q SNP P‐value) for GenomicSEM associations (main) and Q‐Q plot of expected vs. observed –log10 Q SNP P‐values (upper right corners). Solid red line of Manhattan plots denotes genome‐wide significant (GWS) threshold (P < 5 × 10^−8^) and dashed grey line denotes P < 1 × 10^−5^. Purple diamonds represent GWS Q SNPs (n = 21 for alcohol consumption). [file ADB-29-e13365-s003.docx]

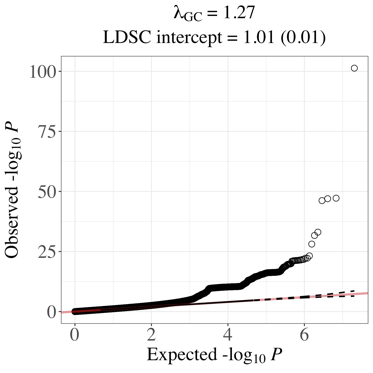

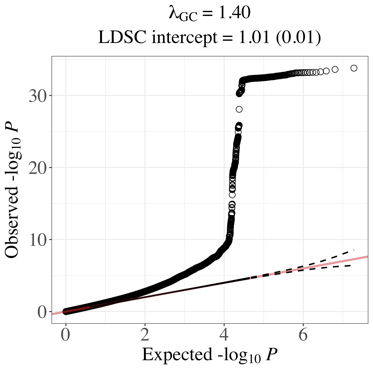

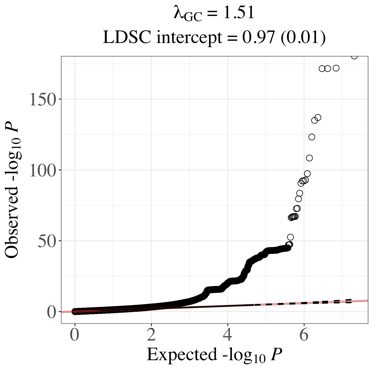


A.

B.

C.

**Figure S1. Q-Q plots for GenomicSEM indicator GWAS meta-analyses***.* These results have not been adjusted for genomic control inflation factors (λ_GC_). **(A)** 23andMe + Linnér et al. risk taking meta-analysis. **(B)** UK Biobank + Million Veteran Program AUDIT-C meta-analysis. **(C)** 23andMe + GWAS & Sequencing Consortium of Alcohol and Nicotine use (GSCAN) drinks per week meta-analysis.


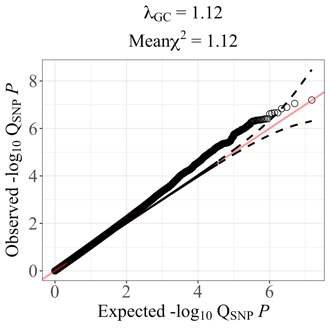

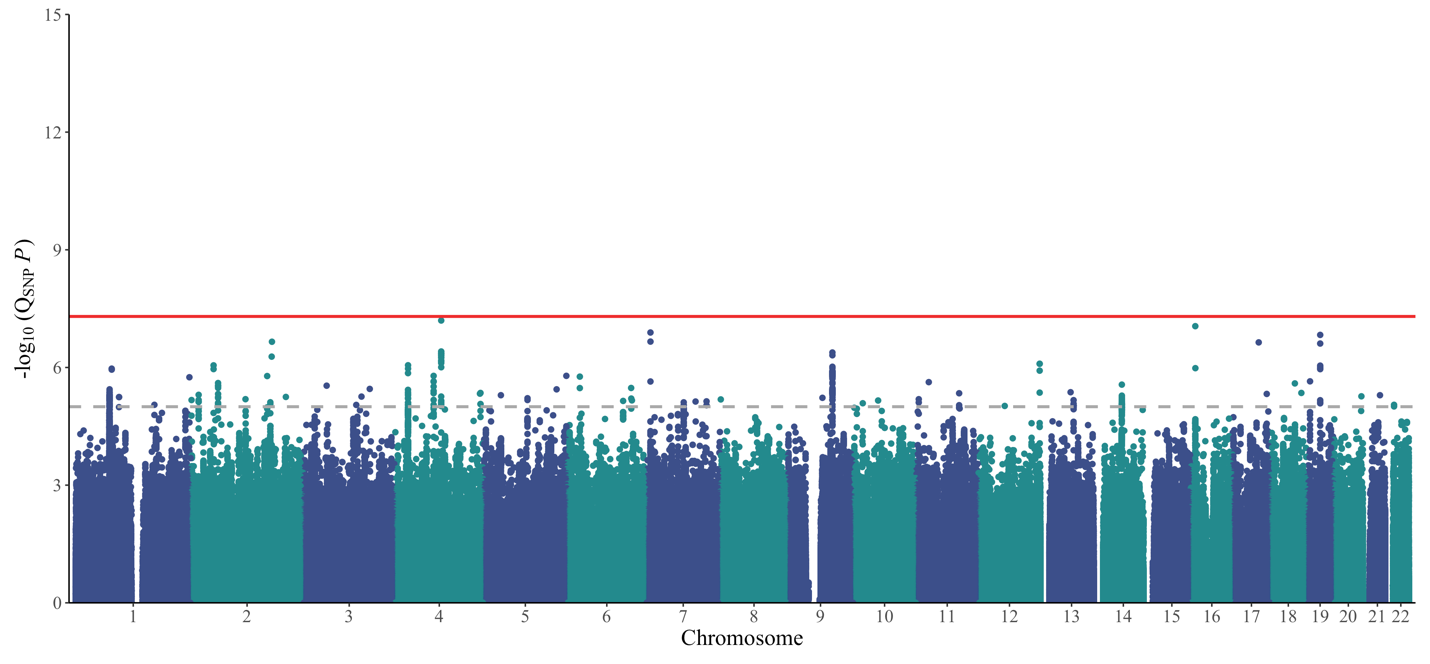


A.


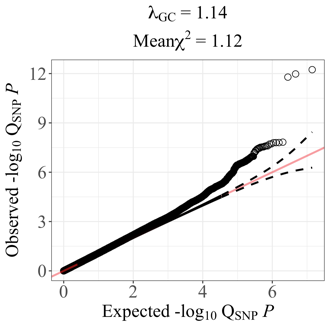


B.


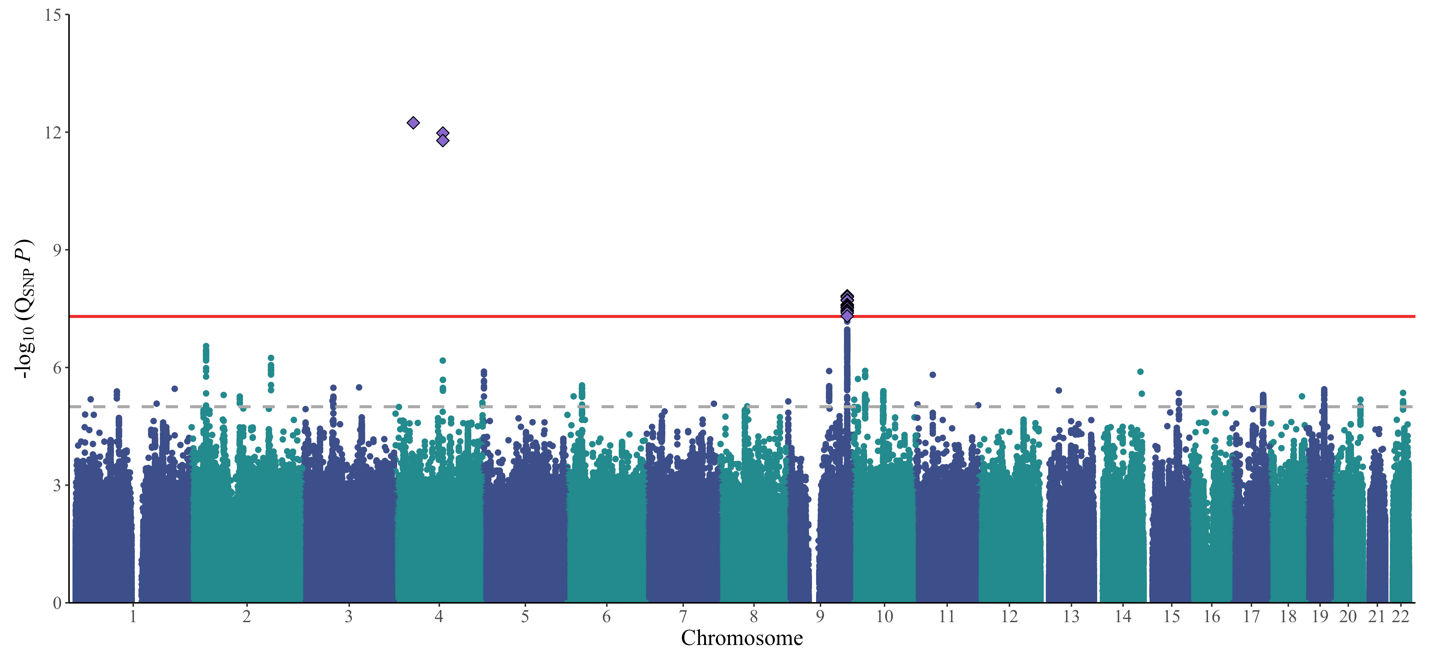


**Figure S2***.* ***Q*_SNP_ analysis of (A) sensation seeking and (B) alcohol consumption.** Manhattan plot of –log10 (two-sided *Q*_SNP_ *P*-value) for GenomicSEM associations (main) and Q-Q plot of expected vs. observed –log10 *Q*_SNP_ *P*-values (upper right corners). Solid red line of Manhattan plots denotes genome-wide significant (GWS) threshold (*P* < 5 × 10^-8^) and dashed grey line denotes *P* < 1 × 10^-5^. Purple diamonds represent GWS *Q*_SNPs_ (*n* = 21 for alcohol consumption).
